# Oblique lateral interbody fusion stand-alone vs. combined with percutaneous pedicle screw fixation in the treatment of discogenic low back pain

**DOI:** 10.3389/fsurg.2022.1013431

**Published:** 2022-10-06

**Authors:** Weiheng Wang, Bing Xiao, Haotian Wang, Junqiang Qi, Xin Gu, Jiangming Yu, Xiaojian Ye, Guohua Xu, Yanhai Xi

**Affiliations:** ^1^ Department of Orthopaedics, Second Affiliated Hospital of Naval Medical University, Shanghai, China; ^2^ Department of Orthopedics, Tongren Hospital, Shanghai Jiao Tong University School of Medicine, Shanghai, China

**Keywords:** discogenic low back pain, OLIF, discography, discoblock, stand alone, percutaneous pedicle screw fixation

## Abstract

**Objective:**

Oblique lateral interbody fusion (OLIF) has unique advantages in the treatment of discogenic low back pain (DBP). However, there are few studies in this area, and no established standard for additional posterior internal fixation. The purpose of this study was to investigate the efficacy of OLIF stand-alone vs. combined with percutaneous pedicle screw fixation (PPSF) in the treatment of DBP.

**Methods:**

This retrospective case-control study included forty patients. All patients were diagnosed with DBP by discography and discoblock. Perioperative parameters (surgery duration, blood loss, and muscle damage), complications, Visual analog scale (VAS), and Oswestry Disability Index (ODI) were assessed. Imaging data including cage subsidence, cage retropulsion, fusion rate, and adjacent spondylosis degeneration (ASD) were analyzed.

**Results:**

There were 23 patients in the OLIF stand-alone group and 17 patients in the OLIF + PPSF group. The mean surgery duration, blood loss, and muscle damage in the OLIF stand-alone group were significantly better than those in the OLIF + PPSF group (*P* < 0.05). However, there was no significant difference in the average hospitalization time between the two groups (*P* > 0.05). There was no significant difference in the VAS and ODI scores between the two groups before surgery (*P* > 0.05), and VAS and ODI scores significantly improved after surgery (*P* < 0.05). The VAS and ODI scores in the OLIF stand-alone group were significantly better than those in the OLIF + PPSF group at 1 month (*P* < 0.05), While there was no significant difference between the two groups at 12 months and last follow up (*P* > 0.05). At the last follow-up, there was no significant difference in cage subsidence, fusion rate, ASD and complication rate between the two groups (*P* > 0.05).

**Conclusion:**

OLIF stand-alone and OLIF + PPSF are both safe and effective in the treatment of DBP, and there is no significant difference in the long-term clinical and radiological outcomes. OLIF stand-alone has the advantages of surgery duration, blood loss, muscle damage, and early clinical effect. More clinical data are needed to confirm the effect of OLIF stand-alone on cage subsidence and ASD. This study provides a basis for the clinical application of standard DBP treatment with OLIF.

## Introduction

Low back pain (LBP) is caused by a group of diseases with dysfunction of the lumbar spine, nerve, or soft tissue (1, 2). The prevalence of LBP in the adult population can be as high as 40%, which seriously affects people's health and medical burden (3). Discogenic low back pain (DBP) accounts for 30% to 40% of all LBP (4). Intravertebral disc disruption (IDD) was first proposed by Crock in 1970 (4), which was caused by the pain receptors in the intervertebral disc without radicular symptoms. DBP was first proposed by Park (5) referring to LBP caused by intervertebral disc degeneration, and nerve root compression was excluded by imaging. The diagnostic criteria for IDD was discography established by the International Association for the Study of Pain (IASP) (6). Discoblock combined with discography can improve the accuracy of DBP diagnosis (7). DBP should adopt a step-by-step treatment plan. Conservative management should be adopted for at least 6 months, and surgery should be considered if symptoms do not resolve (8). In recent decades, lumbar fusion surgery for DBP has become more and more widely (9). The mechanism of lumbar fusion surgery is that discectomy eliminates pain receptors and internal fixation prevents pain from mechanical stress caused by spinal instability. However, the effect of lumbar fusion surgery is controversial. One study showed that there was no significant difference in the relief of LBP between surgery and conservative management (9). The effect of surgical treatment of DBP varies greatly in different reports (10, 11). Regardless of the fusion rate, long-term clinical follow-up showed that there were many patients with LBP with good fusion. Complications after lumbar fusion are also the reasons for poor postoperative outcomes (12, 13). The high misdiagnosis rate of DBP and the damage to the lumbar back muscles and facet joints caused by conventional posterior lumbar spine surgery are the reasons for the poor efficacy of lumbar fusion surgery.

Oblique lateral interbody fusion (OLIF) has special advantages in the treatment of DBP, while there are few related reports so far (14). OLIF is an intervertebral fusion surgery through the retroperitoneal approach, which can better preserve the muscles, ligaments, and bony structures behind the lumbar spine, which greatly reduces the incidence of LBP after surgery (15). Since first reported in 2012, OLIF surgery has been widely used in the treatment of lumbar spinal degenerative diseases (16). It remains controversial whether internal fixation is required for OLIF (17). OLIF stand-alone is characterized by simple operation, short operation time, and no need to change positions during operation (18, 19). The advantages of OLIF stand-alone in the treatment of DBP are that there is no damage to the lumbar back muscles, the intervertebral disc is removed more thoroughly, and the rate of intervertebral fusion is high. However, there is no uniform standard for OLIF treatment of DLBP with or without internal fixation so far.

The purpose of this study was to investigate the efficacy of OLIF stand-alone vs. combined with percutaneous pedicle screw fixation (PPSF) in the treatment of DBP. This is a retrospective case-control study analyzing 40 patients treated with OLIF stand-alone and OLIF combined with posterior pedicle screw fixation (PPSF) by comparing their clinical and radiological outcomes in the treatment of DBP from January 2014 to December 2021 at Shanghai ChangZheng Hospital and Shanghai Tongren Hospital. The conclusions of this study provided a clinical basis for the effectiveness of OLIF in the treatment of DBP. More importantly, it provided a basis for the standardized treatment of DBP with OLIF stand-alone vs. combined with PPSF in clinical practice.

## Research methods

### Study design and patients

This study was a retrospective case-control study, followed up from January 2014 to December 2021 in 40 patients with DBP, who underwent OLIF stand-alone or OLIF + PPSF at Shanghai ChangZheng Hospital and shanghai Tongren Hospital. The study was approved by the ethical committee of the Shanghai Changzheng Hospital and the ethical committee of Shanghai Tongren Hospital. Additionally, the patients provided their written informed consent to participate in this study.

**Figure 1:**
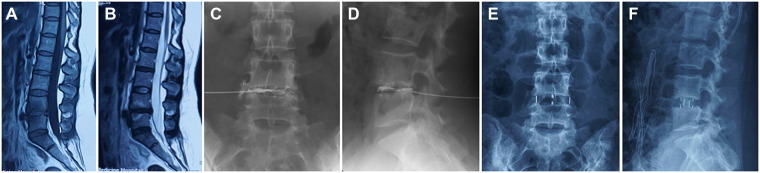
Typical case. Type I Modic changes was characterized by low T1 and high T2 signals in MRI on L4-5 endplate (**A,B**). The patient experienced severe LBP and DBP was a definitive diagnosis by discography and discoblock (**C,D**). The patient underwent OLIF stand-alone (**E,F**).

This study included 40 patients with a definitive diagnosis of DBP, 23 patients underwent OLIF stand-alone, and 17 patients underwent OLIF + PPSF surgery. Demographic data were investigated including gender, age, BMI, bone mineral density (BMD), and surgical segment. The diagnostic criteria for DBP were that the patients had symptoms of LBP diagnosed by imaging, then discography and discoblock were used to further confirm the diagnosis (Figure 1) (20, 21). The inclusion criteria were: (1) DBP was diagnosed by discography and discoblock; (2) conservative treatment failed more than 6 months; (3) no history of lumbar spine surgery at L2-S1; (4) OLIF stand-alone or OLIF + PPSF of L2-S1; (5) more than 12 months follow-up. The exclusion criteria were: (1) LBP without a definitive diagnosis by discography and discoblock; (2) lumbar disc herniation or spinal stenosis; (3) cauda equina syndrome; (4) spinal tumor; (5) paravertebral infection; (6) vertebral fracture; (7) previous surgery for L2-S1; (8) pregnancy, chronic nicotine, alcohol or drug abuse, etc.

### OLIF surgical procedures

OLIF stand-alone group: details of OLIF surgical were performed based on standard procedure (22). After general anesthesia, the patient was placed in the right lateral decubitus position. Under the guidance of fluoroscopy, an oblique skin incision of about 4 cm was made at an anterior 4 cm–6 cm of the center point of the target intervertebral disc. The muscle (external oblique, internal oblique, and transverse abdominis) and retroperitoneal space were bluntly dissected down to the intervertebral disc. Intervertebral space decompression was performed, but direct decompressions were not performed. An OLIF25™ Cage (Medtronic, Sofamor Danek, United States) filled with artificial bone (Aorui, Shanxi, China) was inserted into the intervertebral space. OLIF + PPSF group: The cage placement process was the same as above. After that, the patient was changed to the prone position and PPSF was performed (Johnson / Johnson, United States). Neither group of patients underwent additional laminectomy. Surgery-related parameters (blood loss, surgery duration, hospitalization time, serum levels of creatinine kinase, and complications) were recorded. On the second day after the operation, the patient got out of bed under waist protection, and the waist protection time was less than 3 months. Patients were encouraged to perform low back muscle function exercises (23).

### Clinical and imaging evaluation

The patients received regular follow-ups at 1, 3, and 12 months after the operation and the last follow-up. Patients underwent routine preoperative and postoperative standing anteroposterior (AP)/lateral plain radiographs, computed tomography (CT), and magnetic resonance imaging (MRI). LBP was assessed using a visual analogue scale (VAS). Functional improvement was assessed using the Oswestry Disability Index (ODI). Bone mineral density (BMD) was measured using dual-energy x-ray absorptiometry (DEXA). *T* < −2.5 was defined as osteoporosis. The relationship between the contact surface of the cage and the upper and lower endplates was observed according to the method of Marchi et al. (24). The boundary of the cage beyond the upper or lower endplates was regarded as a settlement (24, 25). Cage subsidence was divided into grades 0-III based on the disc height (DH) immediately after surgery: grade 0, DH decreased by 0%–24%; grade I, DH decreased by 25%–49%; grade II, DH decreased by 50% to 74%; grade III, DH decreased by 75% to 100%. Cage displacement was defined as a posterior movement of the cage ≥3 mm at follow-up compared with the immediate postoperative period. Data were collected before surgery, 1, 3, and 12 months after surgery, and last follow-up. In addition, complications were also recorded, including endplate damage, leg weakness, abdominal distension, and sympathetic chain damage. The fusion rate was evaluated at 1 year and last follow-up. The fusion rate was based on the Bridwell Fusion Grading System (26). Grades I and II were considered successful fusion, and grades III and IV were considered fusion failure. The diagnosis of adjacent spondylosis degeneration (ASD) was based on imaging evaluation. Compared with preoperative, when the DH drops >3 mm, the vertebral body slips forward or backward >3 mm, the intervertebral space is angled posteriorly >5°, and the Pfirrmann grade progresses ≥Level 1 (27). All imaging evaluations were performed independently by two spine surgeons. Further determination was made by a third physician when disagreements arose. Measurements were made by using MicroDicom software.

### Statistical analysis

SPSS 21.0 software (IBM, United States) was used for statistical analysis. Quantitative results were expressed as means ± standard deviation (SD). Between-group comparisons were performed using the independent-samples t-test. Repeated-measurement ANOVA was used for intra-group analysis. The nonparametric test was used for the comparison between groups that did not obey the normal distribution. The counting data such as Cage subsidence and fusion rate were expressed in percentage. The Chi-square test or Fisher exact test was used to analyze counting data. *P* < 0.05 were considered statistically significant.

## Results

### General characteristics and operation data

Forty patients (42 discs) underwent OLIF. Among them, 23 cases (25 segments) underwent OLIF stand-alone, and 17 cases (17 segments) underwent OLIF + PPSF. The patients' general characteristics of the two groups were shown in Table 1. The mean age in the OLIF stand-alone group was 51.81 ± 13.61, and the proportion of males was 26.09%. Among the 25 cages inserted, 2 (8%) at the L2/3 level, 5 (20%) at the L3/4 level, 17 (68%) at the L4/5 level, and 1 (4%) at the L5/S1 level. The average age in The OLIF + PPSF group was 50.24 ± 9.25, and the proportion of males was 35.29%. Among the 25 cages, 3 (17.65%) L3/4 levels, 13 (76.47%) L4/5 levels, and 1 (5.88%) L5/S1 levels. There was no significant difference in mean age, BMI, and osteoporosis rate between the two groups (*P* > 0.05, Table 1). The mean surgery duration and blood loss in the OLIF stand-alone group were significantly better than those in the OLIF + PPSF group (*P* < 0.05). However, there was no significant difference in the average hospitalization time between the two groups (*P* > 0.05, Table 2).

**Table 1:** General characteristics of the patients.

Characteristics	OLIF stand-alone (*n* = 23)	OLIF + PPSF (*n* = 17)	*P*
Mean age, years, ±SD	51.81 ± 13.61	50.24 ± 9.25	0.672
Men, *n* (%)	6 (26.09%)	6 (35.29%)	0.530
BMI	25.36 ± 2.79	26.65 ± 3.12	0.751
Levels of OLIF, *n* (%)	*n* = 25	*n* = 17	0.861
L2/3	2 (8%)	0	
L3/4	5 (20%)	3 (17.65%)	
L4/5	17 (68%)	13 (76.47%)	
L5/S1	1 (4%)	1 (5.88%)	
Osteoporosis, *n* (%)	0	2 (11.76%)	0.174

**Table 2:** Surgical data.

Parameter	OLIF stand-alone (*n* = 23)	OLIF + PPSF (*n* = 17)	*P*
Blood loss (ml)	68.65 ± 23.25	111.5 ± 21.85	<0.0001*
Surgery duration (min)	75.65 ± 30.05	115.29 ± 28.96	0.0002*
Hospitalization time (day)	5.22 ± 2.23	5.29 ± 1.31	0.443

* Statistically significant (*P*-value <0.05).

### Clinical outcomes

**Figure 2:**
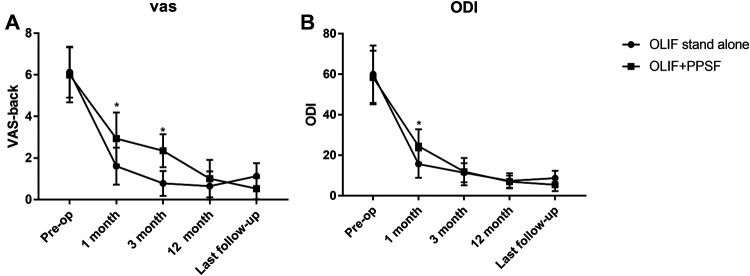
VAS (**A**) and ODI (**B**) score. Comparison between OLIF stand-alone and OLIF + PPSF from pre-operation to last follow-up. *P < 0.05.

There was no significant difference in preoperative serum creatinine kinase level between the two groups (*P* > 0.05). The OLIF + PPSF group had significantly higher postoperative 1 day than the OLIF stand-alone group (*P* < 0.05), but these differences did not persist on postoperative 5 days (*P* > 0.05, Table 3). The clinical outcome of the two groups was shown in Table 3 and Figure 2. There was no significant difference in the preoperative VAS and ODI scores between the two groups (*P* > 0.05). The postoperative VAS and ODI scores of the two groups were significantly improved compared with those before surgery (*P* < 0.05). The VAS scores in the OLIF stand-alone group were significantly better than those in the OLIF + PPS group at the 1 and 3-month follow-up (*P* < 0.05). There was a significant difference in ODI scores between the two groups at 1 month (*P* < 0.05), but no significant difference at 3 months (*P* > 0.05). There were no significant differences in VAS and ODI scores between the two groups at 12 months and the last follow-up (*P* > 0.05). The VAS and ODI scores in the OLIF stand-alone group were better at the last follow-up than at 12 months, but the difference was not statistically significant (Figure 2, *P* > 0.05).

**Table 3:** Clinical outcomes.

Parameter	OLIF stand-alone (*n* = 23)	OLIF + PPSF (*n* = 17)	*P*
**VAS (LBP)**			
Pre-op	5.78 ± 1.20	6.00 ± 1.32	0.592
Post-op 1 month	1.61 ± 0.89	2.94 ± 1.25	<0.001*
Post-op 3 month	0.78 ± 0.60	2.35 ± 0.79	<0.001*
Post-op 12 month	0.65 ± 0.71	1.06 ± 0.90	0.119
final follow-up	1.0 ± 1.0	0.65 ± 0.61	0.206
**ODI**			
Pre-op	60.00 ± 14.14	58.35 ± 13.23	0.710
Post-op 1 month	15.65 ± 6.73	24.47 ± 8.32	0.001*
Post-op 3 month	11.26 ± 3.99	11.24 ± 4.48	0.985
Post-op 12 month	7.391 ± 3.69	6.94 ± 3.01	0.683
last follow-up	8.696 ± 3.60	5.41 ± 3.14	0.004
**Creatinine kinase**			
Pre-op	85.35 ± 34.94	84.82 ± 38.43	0.964
Post-op 1 day	257.70 ± 71.68	340.00 ± 104.50	0.005*
Post-op 5 day	100.17 ± 35.93	110.35 ± 49.47	0.455

* Statistically significant (*P*-value <0.05).

### Cage subsidence and fusion rate

**Figure 3:**
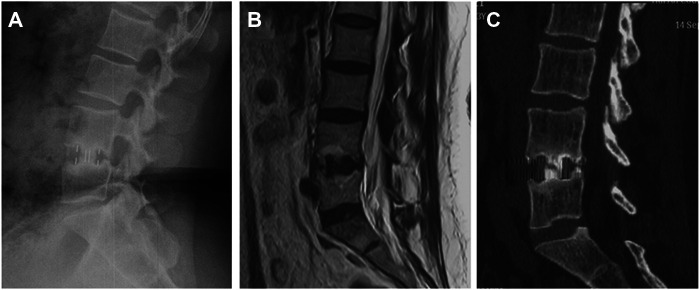
Case example of fusion after Grade I subsidence with no symptoms. 5 years of radiographs (**A**), MRI (**B**), and CT images (**C**) showing fusion obtained after placement of a standard cage despite subsidence occurrence.

Cage subsidence and fusion rate are detailed in Table 4. Cage subsidence occurred in 7 (28%) of 25 segments in the OLIF stand-alone group. Cage subsidence occurred in 2 (11.76%) of 17 segments in the OLIF + PPSF group. According to the Cage subsidence grading method proposed by MARCHI et al. (24), 4 cases of grade 0, 2 cases of grade I, and 1 case of grade II were in the OLIF stand-alone group. 2 case of grade 0 was in OLIF + PPSF group. There was no statistical difference between the two groups in Cage subsidence (*P* = 0.278). An example of fusion at the last follow-up (5 years) in a case of Grade I subsidence is shown in Figure 3, and the patient has no symptoms. There was no cage retropulsion in both groups at the follow-up. At 12 months, the fusion rate of OLIF + PPSF was 94.12% (16/17) and 92% (23/25) in the OLIF stand-alone group. At the last follow-up, the fusion rate of the OLIF + PPSF group was 100.0% (17/17), and the fusion rate of the OLIF stand-alone group was 96% (24/25). There was no statistical difference between the two groups (*P* > 0.999). During the follow-up period, no patients in either group required revision. 2 cases (11.76%) of ASD were found in the OLIF + PPSF group, but the patients had no obvious symptoms and were followed up closely.

**Table 4:** Radiological outcomes.

Parameter	OLIF stand-alone (*n* = 23)	OLIF + PPSF (*n* = 17)	*P*
Fusion rate at 1 year	*n* = 25	*n* = 17	
Grade I	19 (76%)	15 (88.24%)	
Grade II	4 (16%)	1 (5.88%)	
Grade III	2 (8%)	1 (5.88%)	
Grade IV	0	0	
Total fusion rate	23 (92.00%)	16 (94.12%)	>0.999
Fusion rate at last follow-up	*n* = 25	*n* = 17	
Grade I	22 (88%)	17 (100%)	
Grade II	2 (8%)	0	
Grade III	1 (4%)	0	
Grade IV	0	0	
Total fusion rate	24 (96%)	17 (100%)	>0.999
Cage subsidence at last follow-up	*n* = 25	*n* = 17	
Grade 0	4 (16%)	2 (11.76%)	
Grade I	2 (8%)	0	
Grade II	1 (4%)	0	
Grade III	0	0	
Total rate	7 (28.00%)	2 (11.76%)	0.271
ASD	0	2 (11.76%)	0.158

### Complications

The total complication rate was 29.41% (5/17) in the OLIF + PPSF group and 26.09% (6/23) in the OLIF stand-alone group, with no significant difference between the two groups (*P* = 1, Table 5). Intraoperative endplate injury occurred in 2 patients (11.76%) in the OLIF + PPSF group. Leg weakness occurred in 3 cases (13.04%) and 2 cases (11.76%) in OLIF + PPSF group and OLIF stand-alone group, respectively. The patients recovered within 2 weeks after functional exercise. Abdominal distension occurred in 2 cases (8.70%) and 1 case (5.88%) in OLIF + PPSF group and OLIF stand-alone group, respectively. Transient sympathetic nerve injury and leg numbness occurred in 1 case (4.35%) in OLIF stand-alone group. At the 3 month follow-up, the patient's symptoms disappeared.

**Table 5:** Complications outcomes.

Parameter	OLIF stand-alone (*n* = 23)	OLIF + PPSF (*n* = 17)	*P*
Endplate damage	0	2 (11.76%)	0.174
Leg weakness	3 (13.04%)	2 (11.76%)	>0.999
Abdominal distension	2 (8.70%)	1 (5.88%)	>0.999
Sympathetic chain damage	1 (4.35%)	0	>0.999
total	6 (26.09%)	5 (29.41%)	>0.999

## Discussion

OLIF is characterized by minimally invasive, high fusion rate, and low complications (28, 29). It has been widely used in spinal degenerative scoliosis (30, 31), spondylolisthesis (18), spinal stenosis (32), ASD (33, 34), and DBP (14), and has achieved good clinical effects. This study further confirmed the well early and mid-term effects of OLIF in the treatment of DBP. OLIF stand-alone and OLIF + PPSF are both safe and effective in the treatment of DBP, and there is no significant difference in the long-term clinical effect. OILF stand-alone can significantly reduce the surgery duration, blood loss, and intraoperative muscle damage, and can significantly improve the early postoperative clinical effect. In addition, our study also found that the ASD rate was 11.76% and 0%, and Cage subsidence was 11.76% and 28.00% in the OLIF + PPSF group and OLIF stand-alone group, respectively. Limited by the number of cases, there was no statistical difference between the two groups. OLIF stand-alone may reduce ASD and increase Cage subsidence, which needs more clinical data to confirm. OLIF stand-alone and OLIF + PPSF are both safe and effective in the treatment of DBP, and there is no significant difference in the long-term clinical and radiological outcomes. Our long-term study case observation found that in OLIF stand-alone, despite cage subsidence, the patient had no obvious clinical symptoms with definite interbody fusion (Figure 3).

In recent decades, with the widespread application of lumbar fusion, more and more patients with chronic low back pain have received lumbar fusion (35). However, the effects of lumbar fusion in the treatment of DBP vary widely among different reports. One study showed that the 1-year success rate was only 33% for surgical treatment of DBP (10). Regardless of the fusion rate, long-term clinical follow-up showed that there were many patients with LBP with good fusion. Lumbar fusion surgery is considered the standard procedure for the treatment of DBP. However, due to the large differences in the definition and diagnostic criteria of DBP, different kinds of literature have various inclusion and exclusion criteria for patients (36). The diagnostic criteria and surgical indications for DBP vary widely in different kinds of literature, resulting in differences in postoperative outcomes. Postoperative LBP may originate from complications after lumbar fusion (fusion failure, cage subsidence, intraoperative low back muscle injury, and ASD). Due to these, outcomes of lumbar fusion surgery vary widely. A clear diagnosis of DBP and strict control of surgical indications are the keys to ensuring the effect of surgical treatment. The diagnostic criteria for IDD is discography established by the International Association for the Study of Pain (IASP). Positive discography criteria are consistent pain response and no consistent pain in at least one adjacent disc (6). Discography is characterized by high sensitivity and poor specificity (37, 38). Discoblock combined with discography can improve the accuracy of DBP diagnosis (7). A long-term clinical study showed that discography can significantly increase the degree of disc degeneration (39). Our previous study also showed that needle diameter, type, and volume of contrast agent with discography had a significant effect on intervertebral disc degeneration (40). The following was our experience in the diagnosis of LBP with discography. First, the indications for discography should be strictly controlled. Second, the operation procedure of discography should be strictly standardized. During the operation of discography, a small puncture needle and a less dose of contrast agent should be used. Third, when the discography is positive, discoblock should be performed to improve the efficiency of diagnosis. Fourth, the adjacent segment negative control was not performed due to a significant increase in disc degeneration. Due to strict control of the diagnostic criteria and surgical indications for DBP, only forty patients diagnosed with DBP and who underwent OLIF from January 2014 to December 2021 were included in the study. To our knowledge, this is the largest number of patients included in the study of OLIF treatment of DBP.

OILF stand-alone has special advantages in the treatment of DBP. The surgical approach of OLIF is performed from the side of the lumbar spine (16). Compared with traditional posterior lumbar fusion surgery, OLIF hardly damages the posterior paravertebral muscles, ligaments, and facet joints, which greatly reduces the incidence of postoperative LB. It is critical for postoperative symptomatic improvement in DBP (41). OLIF is characterized by more thorough removal of the interstitial disc and placement of a larger Cage into the intervertebral space. Through more thorough treatment of the intervertebral space, it helps to destroy the pain receptors in the diseased intervertebral disc in DBP. In addition, the Cage used in OLIF was relatively large. Previous biomechanical studies have shown that after the Cage was placed into the intervertebral space, the annulus fibrosus and the anterior and posterior longitudinal ligaments were stretched so that the Cage can be stabilized immediately after the operation (42). Our study showed that OLIF stand-alone treatment of DBP can reduce operation time, blood loss, and low back muscle damage, and improve early postoperative outcomes.

Although OLIF stand-alone surgery has many advantages, the problem of postoperative cage subsidence cannot be ignored. Previous clinical studies and Meta-analysis showed that the Cage subsidence rate after lateral anterior stand-alone surgery was about 18% (43, 44). However, the present study found that the postoperative Cage subsidence rates in the OLIF stand-alone and OLIF + PPSF groups were 28% and 11.76%, respectively, which was consistent with previous literature reports. The study of TEMPEL et al. (45) found that postoperative Cage subsidence was an important predictor of reoperation after stand-alone surgery. Not all cases of Cage subsidence require revision surgery. Reoperation is required only in cases of severe Cage subsidence with symptoms of severe nerve compression. There is currently no clinical standard about the degree of Cage subsidence that requires supplemental posterior pedicle screw fixation. During our follow-up period, no revision cases were found. Although cage subsidence was high in OLIF stand-alone, it had no significant effect on long-term fusion rate and clinical efficacy. The clinical efficacy at the last follow-up after OLIF stand-alone decreased, but the difference was not statistically significant. This may be caused by post-operative Cage subsidence. Additional PPSF can maintain the clinical efficacy after OLIF, prevent Cage subsidence, and may reduce the risk of revision. Modic changes and endplate sclerosis were highly correlated with cage subsidence. Cage sedimentation rates were lower after OLIF stand-alone in patients with endplates with type III Modic changes, hardened endplates, and flat endplate morphology (46). Osteoporosis (47), age/gender, (24), preoperative CT value (Hounsfield unit, HU) measured in the endplate area (48), and intraoperative endplate injury (19) are risk factors for postoperative cage subsidence. Therefore, in the treatment of DBP with OLIF, additional PPSF is recommended for elderly women, patients with osteoporosis, and intraoperative endplate damage. PPSF may increase ASD has been reported in the literature (19). The reason for this may be that the posterior lumbar spine surgery damages the lower back muscles and adjacent facet joints (49, 50), increasing ASD. Limited by the number of cases, our study did not find a statistically significant reduction in ASD with OLIF stand-alone. The effect of OLIF stand-alone on cage subsidence and ASD needs more clinical data to confirm.

There were some limitations in this study. First, this study was a retrospective case-control study. Unavoidably, there was a certain degree of selection bias may impact the results. Second, the sample size of this study was limited, and a larger sample size is needed to confirm this conclusion. Therefore, the results of this study needed to be further verified by multi-center randomized double-blind study data.

## Conclusion

Both OLIF stand-alone and OLIF combined with PPSF were safe and effective in the treatment of DBP, and there was no significant difference in long-term clinical and radiological outcomes. OLIF stand-alone has the advantages of shorter operation time, less blood loss, less muscle damage, and better early clinical efficacy. The effect of OLIF stand-alone on cage subsidence and ASD needs more clinical data to confirm. For patients who are osteoporotic and have intraoperative endplate damage, OLIF combined with PPSF may be superior to monotherapy.

## Data Availability

The original contributions presented in the study are included in the article/Supplementary Material, further inquiries can be directed to the corresponding author/s.
